# Evaluation of genetic consequences of stocking on the southern‐margin populations of white‐spotted charr

**DOI:** 10.1002/ece3.70140

**Published:** 2024-08-09

**Authors:** Taro Masuda, Yoshiko Shimono, Daisuke Kishi, Itsuro Koizumi

**Affiliations:** ^1^ Laboratory of Marine Biology, Division of Applied Biological Science, Faculty of Agriculture Setsunan University Hirakata, Osaka Japan; ^2^ Laboratory of Weed Science, Graduate School of Agriculture Kyoto University Kyoto Japan; ^3^ Gero Branch, Gifu Prefectural Research Institute for Fisheries and Aquatic Environments Gifu Japan; ^4^ Faculty of Environmental Earth Science Hokkaido University Sapporo Hokkaido Japan

**Keywords:** ancient lake, conservation, distribution margin, gene flow, genetic introgression, Lake Biwa, local population, stocking, white‐spotted charr

## Abstract

Coldwater‐adapted freshwater fishes, especially their populations along warm‐range margins, are most vulnerable to the climate oscillations associated with global warming. Stocking is a major strategy for avoiding the extinction of these species. However, while stocking can reverse the decline of isolated populations, it may also result in a loss of genetic diversity in the native local population due to the introgressive replacement of hatchery genes. To plan an adequate strategy for conserving locally adapted populations, the genetic impacts of stocking on native lineages should be evaluated from small river branches to wide‐ranging drainage areas. We investigated the population genetic structure of white‐spotted charr (*Salvelinus leucomaenis*) within its southern range (Lake Biwa basin, Japan). By applying genome‐wide SNP analysis to the population's genetic structure, we assessed the extent of genetic introgression resulting from stocking. White‐spotted charr in the Lake Biwa watershed constitutes a distinctive genetic group, within which apparent genetic differentiation was observed. The hatchery‐reared fish line commonly used for supplementation stocking in the catchment was discernable from the native population, enabling us to analyze genetic introgression across the entire drainage area. Admixed individuals resulting from hatchery introgression were observed in most of the stocked sites that showed relatively high heterozygosity and nucleotide diversity. However, their genetic differentiation was much lower than that of native populations. The supplementation history as well as the road availability contributed substantially to the introgression of hatchery genes. Populations with the native genetic structure remained in the upstream regions of the tested rivers. However, their heterozygosity and nucleotide diversity were low when compared with that of the populations with hatchery supplementation. Our results shed light on the genetic impacts of stocking on isolated native populations and suggest that conventional supplementation methods cannot preserve a unique biodiversity in the distribution margin.

## Graphical abstract

White‐spotted charr in the Lake Biwa basin has a distinct genetic structure. The native populations remain only above the dam where individuals are free from genetic introgression of stocking.

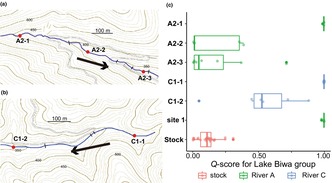

## 1 INTRODUCTION

The freshwater ecosystem has extreme biodiversity relative to its tiny share of Earth's total aquatic area. Although habitable freshwater occupies only 0.009% of the Earth's water volume, it harbors at least 40% of all identified ray‐finned fish species (Dudgeon et al., 2006; Miller, 2021). During the current Anthropocene period, freshwater biodiversity is the overriding conservation priority. Indeed, the United Nations designated the years 2005–2015 as the International Decade for Action “Water for Life” (https://www.un.org/waterforlifedecade/) and 2011–2020 as the United Nations Decade on Biodiversity (https://www.cbd.int/2011‐2020/) (Rands et al., 2010). One reason for the importance of freshwater biodiversity is that the richness of species diversity is highly correlated with freshwater fishery yields, and thus species richness can underpin fisheries productivity in a mutually beneficial relationship (Brooks et al., 2016). Biodiversity conservation for freshwater fishes should be adopted not only for threatened species but also for locally differentiated and/or adapted regional populations that will be incipient species for further speciation (Dudgeon et al., 2006; Hasselman et al., 2013; Ricklefs, 2004).

Stocking of hatchery‐reared fish is one of the most frequently used strategies for reducing the risk of the decrease or extinction of native populations of freshwater fishes (Naish et al., 2007). Recently, many sophisticated studies have reconsidered the effectiveness of stocking to maintain fish resources (Radinger et al., 2023; Terui et al., 2023). Conventional supplementation strategies for enhancing fishery production and recovering declining populations are sometimes less effective than expected. Moreover, supplemented individuals can be a long‐term threat to wild populations and native whole‐stream fauna if genetic interactions occur between native and released captive‐breeding individuals (Araki et al., 2008; Terui et al., 2023; Willoughby & Christie, 2019). Ordinary stocking practices using domesticated fish can give rise to unexpected results, such as a reduction in genetic differentiation between populations, leading ultimately to a loss of locally adapted populations with superior fitness in regional environments (Araki & Schmid, 2010; Willoughby & Christie, 2019). Hence, to preserve natural genetic structures with locally adapted variations, it is paramount to understand how human‐mediated supplementation affects native assemblages.

Charr, or *Salvelinus*, is a stenothermal coldwater specialist in the salmonid family (Dunham et al., 2008; Selong et al., 2001). Since charr have a long history of interaction with human beings as a good target for fisheries and recreation fishing in the high latitudes of the Northern Hemisphere (Kershner et al., 2019), many attempts to supplement captive breeding individuals have been carried out (Kitano, 2004; Lecomte et al., 2013). Genetic introgression from captive‐born to wild individuals has been investigated, mainly in brook charr (*S. fontinalis*) (Lamaze et al., 2012; Lehnert et al., 2020; Marie et al., 2010), and in some cases, these analyses have incorporated landscape conditions (Bruce et al., 2020; Hargrove et al., 2022; Létourneau et al., 2018). Some of these studies suggest that genetic introgression from stocks to native individuals occurs and may be dependent on supplementation densities (Lamaze et al., 2012; Lehnert et al., 2020; Marie et al., 2010), while others suggest that the problem is not as serious as originally thought (Lehnert et al., 2020; White et al., 2018). Finally, one study has suggested that the genetic traces of stocking will diminish and that the wild genetic state will be restored after stocking is stopped (Létourneau et al., 2018).

**FIGURE 1:**
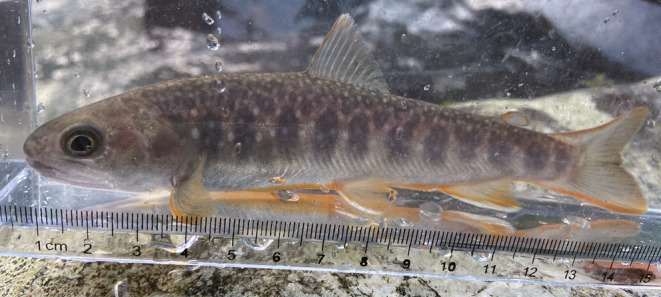
A native white‐spotted charr from the Lake Biwa drainage river.

**FIGURE 2:**
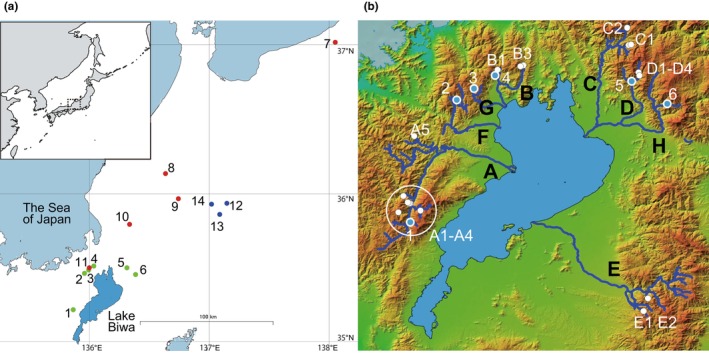
Maps of sampling sites. (a) Map of the Reference sampling sites. Color codes of sampling sites (circles) are depicted as follows: The Sea of Japan watershed, the Pacific Ocean watershed, and the Lake Biwa watershed are shown in red, blue, and green, respectively. The number of each sampling site corresponds to that in Table 1. This map is based on the Digital Map published by the Geospatial Information Authority of Japan. (Inset) Map of the Japanese archipelago and surroundings. The black dotted rectangle shows the location of (a). (b) Map of sampling sites in rivers flowing into Lake Biwa. Tested rivers are depicted by blue lines labeled A–E. Rivers F–H contain the reference sites 2, 3, and 6 in (a), respectively. Reference sites are indicated by blue spots encircled in white, while white spots indicate tested sites. Reference sites no. 1, 4, and 5 correspond to the tested sites A1, B2, and D4, respectively (Table 2). This map is based on the Digital Elevation Map published by Geospatial Information Authority of Japan.

White‐spotted charr (*S. leucomaenis*) (Figure 1) is a southern species within the genus and is predominantly distributed in the Northwestern Pacific regions. The western part of Honshu Island, Japan, is designated as the southern distribution border of this species (Dunham et al., 2008). Several non‐anadromous subspecies or local genetic groups are seen around the southwestern range, where white‐spotted charr typically remain landlocked upstream in rivers. Two major such subspecies, *S. l. pluvius* (distributing in the Sea of Japan watershed) and *S. l. japonicus* (the Pacific watershed), have historically been assumed based on their distinct appearances, and recent single nucleotide polymorphism (SNP)‐based genotyping clearly differentiated these two subspecies from each other (Masuda et al., 2023; Yamamoto et al., 2023). In addition, white‐spotted charr inhabiting the headwater branches in Lake Biwa (670.4 km^2^, the largest lake in Japan) watershed (Figure 2) can be genetically distinguished from the other two subspecies. According to the biogeographical distribution, this lineage is considered the southern border genetic group within its continuous distribution range, except for some small and isolated populations in the Kii Peninsula (Sato & Harada, 2008). Supplementation of domesticated white‐spotted charr is also conducted by regional fishery cooperatives in the rivers of the Lake Biwa catchment, using the stocked strain reared at a single hatchery in common (Kikko, Kai, et al., 2008).

Our objective in this study was to investigate how the stocking of domesticated individuals genetically influences differentiated regional groups, using the white‐spotted charr populations in the Lake Biwa watershed as a model. Lake Biwa, located in the western part of Honshu Island, is an ancient lake, dating back approximately 4 Myr, in the temperate region of Asia. It settled in its current location approximately 0.4 million years ago, after environmental transition since its formation (Rossiter, 2000). Due to its historical environmental transition and isolation, Lake Biwa has a particular biodiversity with unique fauna and flora. The fish fauna in the Lake Biwa drainage area consists primarily of species typical of temperate zones (Tabata et al., 2016). However, coldwater‐adapted salmonids such as white‐spotted charr are also seen in this drainage area. White‐spotted charr populations inhabiting the river upstream in the Lake Biwa basin are generally small and isolated because most of the habitats are fragmented by natural or artificial constructs such as erosion control dams (Kikko, Kuwahara, et al., 2008). Under these circumstances, the genetic diversity of these populations tends to decrease (Carim et al., 2017), whereas the genetically differentiated regional populations are maintained owing to the lack of gene flow from stocked individuals (Morita & Yamamoto, 2002).

To formulate a practical conservation plan for these marginal populations, knowledge of the genetic influence of stocking as well as population genetics and biogeography of the species is indispensable (Olden et al., 2010). It is necessary to conduct fine‐scale analyses focusing on the genetic structure of fish inhabiting small tributaries and specific sections within them. Most of the previous studies for charr genera that focus on the interbreeding between native and domesticated fish have mainly used wide‐range drainage areas as experimental fields (Brookes et al., 2022; Lamaze et al., 2012; Létourneau et al., 2018; Marie et al., 2010). In contrast, far fewer studies have been performed to analyze such genetic effects on the populations inhabiting the headwater branches (Bruce et al., 2020; Bruce & Wright, 2018; Humston et al., 2012). Although the dispersal of domesticated fish, along with their reproduction and interbreeding with native individuals have been observed, the occurrence of introgressive hybridization between wild and domesticated fish largely depends on many factors such as the natural landscape and water quality that is affected by the human activity (Bruce et al., 2020; Humston et al., 2012). The multiplicity and genetic complexity in the stocked strains would make analysis of the genetic structure difficult.

White‐spotted charr populations in the Lake Biwa watershed possess two key strengths that make them an ideal candidate for evaluating the genetic impact of stocking on native populations at a whole‐drainage level: (1) populations within the wide‐range catchment belong to a distinct regional genetic group; and (2) an established strain has been used for supplementation in each branch of the catchment (Kikko, Kai, et al., 2008). Further, by using isolated landlocked populations that are basically lacking in gene flow with downstream or other branches, we can directly evaluate the human‐mediated influence on native populations. In this study, we first sought to characterize the population genetic structure of both native and hatchery‐reared strains in the Lake Biwa basin based on NGS‐based SNP analysis using MIG‐seq (multiplexed inter‐simple sequence repeat (ISSR) genotyping by sequencing) (Suyama & Matsuki, 2015). Next, we analyzed samples from five river systems within the Lake Biwa drainage area to assess the introgression of the hatchery line in relation to the landscape conditions. Finally, we further examined the population genetic structure of the Lake Biwa genetic group to elucidate its phylogeographical aspects, since this information is a prerequisite to provide appropriate conservation strategies. The present study aims to inform management decisions for the conservation of endangered local populations worldwide.

## 2 MATERIALS AND METHODS

### 2.1 Study sites and sample collection

Lake Biwa and its drainage rivers constitute a large water system (Figure 2) that nurtures many endemic species (Tabata et al., 2016). To analyze the genetic structures of testing populations from the Lake Biwa drainage and hatchery individuals, 14 sites in three major watersheds—the Pacific, the Sea of Japan, and Lake Biwa—were set as reference sites where native populations lived (Masuda et al., 2023) (Figure 2a, Table 1). White‐spotted charr inhabiting rivers around the sampling sites are all assumed to be landlocked populations because our sampling sites were upstream branches that are isolated physically by dams, and populations with anadromous life history are generally distributed to the northern part of Honshu Island or further north (Dunham et al., 2008). Samples were collected from 2020 to 2022, mainly by angling. A backpack electrofisher instrument (LR‐24; Smith‐Root Inc.) was also used at some sites. We purchased hatchery‐reared white‐spotted charr from Samegai Salmon Farm, where fish for supplementation in the whole Lake Biwa drainage area are bred. The stocked individuals in the tested sites (all in the Lake Biwa drainage area) originated from this hatchery. The adipose fin of each individual from the sampling sites was dissected and stored at −25°C until use.

**TABLE 1:** Information and population genetic statistics of reference samples.

River system	Pop. no.	Altitude (m)	*N*	*N*_a_	*N*_E_	*P*_i_	*H*_O_	*H*_E_	*G*_IS_
Biwa	1	419	5	1.103	1.046	0.03371	0.035	0.034	−0.029
Biwa	2	420	7	1.243	1.110	0.07735	0.089	0.076	−0.167
Biwa	3	546	7	1.196	1.082	0.05947	0.072	0.058	−0.245
Biwa	4	318	12	1.192	1.108	0.06815	0.075	0.068	−0.100
Biwa	5	314	8	1.218	1.084	0.06006	0.066	0.059	−0.118
Biwa	6	372	13	1.192	1.104	0.06421	0.066	0.064	−0.035
Japan Sea	7	468	7	1.465	1.226	0.1553	0.156	0.155	−0.003
Japan Sea	8	804	10	1.506	1.247	0.1635	0.157	0.164	0.041
Japan Sea	9	707	9	1.360	1.183	0.1193	0.123	0.119	−0.030
Japan Sea	10	371	6	1.317	1.169	0.1141	0.117	0.114	−0.026
Japan Sea	11	524	10	1.424	1.230	0.1481	0.160	0.147	−0.085
Pacific	12	839	10	1.233	1.107	0.07352	0.074	0.073	−0.008
Pacific	13	794	8	1.194	1.087	0.05974	0.071	0.059	−0.207
Pacific	14	1121	8	1.203	1.086	0.05943	0.066	0.059	−0.125
Stock	S	NA	29	1.671	1.234	0.1567	0.153	0.158	0.033
			149						

*Note*: Reference populations were obtained from the 14 sites of three major river systems: Lake Biwa (Biwa), the Sea of Japan (Japan Sea), and the Pacific (Pacific). “Stock” means the hatchery‐reared population used for supplementation in the Lake Biwa watershed. The genetic diversity indices of *N*_a_, *N*_E_ (number of alleles and effective number of alleles), *P*_i_ (nucleotide diversity), *H*_O_, *H*_E_ (observed and expected heterozygosity), and *G*_IS_ (inbreeding coefficient) are shown. The altitude (m) of each sampling site is also shown.

Populations from 19 sites along the branches of five rivers (Rivers A–E) flowing into Lake Biwa were selected as testing populations (Table 2 and Figure 2b). Three Lake Biwa drainage rivers which provided only reference samples were designated as Rivers F, G, and H. Among these rivers, River B, F, and G are not under the control of any fishery cooperatives, while other rivers (Rivers A, C–E, and H) were managed by the fishery cooperatives for each river. The headwater branches with various stocking histories, which are indicated by a + or − in the “supplementation history” column of Table 2, were selected as sampling sites. River A had nine sampling sites, including three sites (A2‐1, A2‐2, and A2‐3) from the upper, middle, and lower stretches of the same branch, and two sites (A3‐1 and A3‐2) from the upper and lower stretches of another branch. Similarly, Rivers B and C contained four and three sites, respectively. Among these sites, B3‐1 and B3‐2 were from one branch, and sites C1‐1 and C1‐2 were from another. Despite their proximity, sites within the same branch were physically separated by dams.

**TABLE 2:** Information and population genetic statistics of tested samples.

Pop. no.	Altitude (m)	*N*	*N*_a_	*N*_E_	*P*_i_	*H*_O_	*H*_E_	*G*_IS_	Supplementation history	Road availability	Isolation by dams or falls
A1 (1)	419	7	1.103	1.046	0.03371	0.03500	0.034	−0.029	−	−	+
A2‐1	453	4	1.202	1.107	0.07972	0.081	0.08	−0.015	−	−	+
A2‐2	383	5	1.433	1.221	0.1563	0.154	0.157	0.018	+	+	−
A2‐3	345	8	1.598	1.277	0.1887	0.203	0.187	−0.082	+	+	−
A3‐1	516	3	1.511	1.309	0.2227	0.235	0.22	−0.069	+	+	−
A3‐2	337	3	1.419	1.281	0.1936	0.161	0.202	0.2	+	+	−
A4	719	5	1.179	1.094	0.06588	0.083	0.063	−0.309	−	−	+
A5	346	14	1.120	1.054	0.03571	0.045	0.035	−0.274	−	−	+
A6	427	8	1.162	1.075	0.05135	0.063	0.05	−0.256	−	−	+
B1	376	5	1.341	1.175	0.1234	0.139	0.121	−0.149	−	+	+
B2 (4)	318	12	1.192	1.108	0.06006	0.075	0.068	−0.1	−	−	+
B3‐1	518	5	1.283	1.155	0.1070	0.113	0.106	−0.062	−	−	+
B3‐2	370	6	1.254	1.103	0.07520	0.085	0.074	−0.151	−	+	−
C2	429	7	1.243	1.106	0.07658	0.072	0.077	0.067	−	−	+
C1‐2	352	6	1.480	1.255	0.1715	0.189	0.169	−0.116	+	+	−
C1‐1	426	7	1.135	1.065	0.04574	0.053	0.045	−0.169	−	−	+
D1	449	3	1.284	1.178	0.1272	0.128	0.127	−0.012	−	−	+
D2	424	7	1.259	1.121	0.08493	0.093	0.084	−0.107	−	+	−
D3	377	3	1.167	1.073	0.0600	0.048	0.063	0.235	−	+	−
D4 (5)	314	8	1.218	1.084	0.06006	0.066	0.059	−0.118	−	−	+
E1	445	10	1.440	1.183	0.1261	0.14	0.125	−0.122	+	−	−
E2	504	14	1.536	1.180	0.1247	0.126	0.125	−0.012	+	−	−
		150									

*Note*: Populations A1, B2, and D4 were also used as reference samples (population numbers as reference samples are shown in brackets). In addition to genetic diversity metrics, the status of each site, supplementation history, road availability, and isolation from the downstream supplementation site by dams and/or falls are also shown.

Since River B was not managed by a fishery cooperative, the River B water system, including its branches, can be considered a nonstocking river. In contrast, River E underwent substantial annual supplementation in almost every major and minor branch, rendering it the most heavily stocked river system in this study. Rivers A, C, and D had certain branches or areas that the fishery cooperatives did not stock. We selected sampling sites along each river, including points both with and without artificial supplementation. The supplementation history is shown in Table 2, together with the altitude and road availability at each sampling site. When a sampling site was more than 50 m from a road for vehicles, we defined the road availability as “negative (−)”. Additionally, in Table 2, the term “isolation” is used when a dam isolates a site from downstream stocked areas.

### 2.2 MIG‐seq procedure

Genomic DNA was extracted from each sample using MagExtractor Genome (Toyobo, Osaka, Japan). The MIG‐seq procedure was carried out according to the method described previously (Masuda et al., 2023), which is a slightly modified version of the original protocol (Suyama & Matsuki, 2015). After the first and second polymerase chain reactions (PCR), the second sets of PCR products were pooled, and fragments of approximately 300–800 bp were selected using SPRIselect (Beckman Coulter, Brea, CA, USA). The size distribution and concentration of the collected fragments were analyzed by an Agilent 2100 Bio‐analyzer (Agilent, Santa Clara, CA, USA) and a Qubit 4 fluorometer (Thermo‐Fisher, Waltham, MA, USA), respectively. After library preparation, sequencing was conducted on a MiSeq platform (Illumina San Diego, CA, USA) using the MiSeq Reagent Kit v3 (150 cycles) (Illumina).

### 2.3 SNP detection and genetic structure analysis

Low‐quality reads and primer regions were removed from the dataset using the Trimmomatic program version 0.36 (Bolger et al., 2014). Quality‐based trimming was performed using a 4 bp sliding window and an average minimum quality score of 15. The allowed mismatch number, palindrome clip threshold, and simple clip threshold were set as 2, 20, and 10, respectively. Minimum and maximum length thresholds for the sequence reads were set as 74 and 75, respectively. Samples with less than 10,000 reads were excluded from the following population genomic analyses. The program package Stacks version 2.54 (Catchen et al., 2013) was used to assemble reads and identify polymorphic loci. The Stacks pipeline was running based on the script “denovo_map.pl” (https://catchenlab.life.illinois.edu/stacks/comp/denovo_map.php). Loci from each fish were built from the obtained reads using the “ustacks” program, setting the minimum depth of coverage required to create a stack (*m*) at 3, the maximum distance in nucleotides allowed between stacks (*M*) at 2, and the maximum distance allowed to align secondary reads to primary stacks (*N*) at 4. A catalog of all loci from each sample fish was created using the “cstacks” program, with the number of mismatches allowed between loci (*n*) set at 2. Loci from each individual were matched against the catalog using the “sstacks” program. The SNPs were retrieved using the “populations” program with the following parameters: a minimum minor allele frequency (min_maf) of 0.01, a minimum percentage of a locus shared within a population (*r*) at 70%, and all samples being considered as a single population (*p* = 1), initially. All analyses in Figure 2 were performed as *p* = 1. For the subsequent analyses, calculation of population genomic statistics, pair‐wise *F*_ST_, and STRUCTURE analyses in Figure 6, numbers of populations were set corresponding to the numbers actually used. The option (‐‐max‐obs‐het) was used in the population analysis to specify a maximum observed heterozygosity of 0.5. To select one SNP per a locus, the option (‐‐write‐random‐snp) was used.

### 2.4 Population genetic structure analysis

Summary statistics of within‐population genetic variation, including the observed (*H*_O_) and expected (*H*_E_) heterozygosity, nucleotide diversity (*P*_i_), fixation index (*F*_IS_), and effective number of alleles (*N*_e_), were calculated using GenoDive 3.04 (Meirmans, 2020).

Inferred ancestry for individuals from each sampling location was determined by a Bayesian clustering approach implemented with the program STRUCTURE version 2.3.4 (Falush et al., 2003) using the polymorphism information of the samples output by the “populations” program. This method was carried out to assess the number of genetic clusters in the data. The number of genetic clusters (*K*) was set to a range of 1–8. The simulations were run 10 times for each value of *K* (1–8) using burn‐in periods of 100,000 and 100,000 Markov chain Monte Carlo (MCMC) replications. The admixture model and the allele frequency correlated models were used (Falush et al., 2003). The most likely value of *K* was defined based on the rate of change in the log probability of data between successive *K* values (Evanno et al., 2005). The program Pophelper version 2.3.1 (Francis, 2017) was used to average the *Q* score calculated by STRUCTURE and to generate bar plots.

### 2.5 Multiple regression analysis

The averaged *Q*‐score of the Lake Biwa genetic cluster for each tested and reference population from the Lake Biwa catchment was calculated and collectively set as a single response variable. Altitude, history of stocking, road availability, and presence of a dam that isolated the sampling site from the downstream were set as explanatory variables (Table 2). Multiple regression analysis was done using R (version 4.2.2).

### 2.6 Analyses of isolation by distance

Genetic distances for all population pairs were calculated as [*F*_ST_/(1 − *F*_ST_)] (Rousset, 1997). The genetic distances among native populations (the reference sites of the Lake Biwa watershed and populations A2‐1, A4, A5, A6, C1‐1, D3, and D4) and stocked populations were plotted separately against geographic distance. The direct geographic distances were calculated from the longitude and latitude of each pair using the website https://www.nhc.noaa.gov/gccalc.shtml. The correlation between genetic and geographic distances was tested with a Mantel test with 10,000 randomizations, using the vegan package (ver. 2.5‐7) (https://rdrr.io/cran/vegan/) for R.

## 3 RESULTS

### 3.1 Population structure of native and hatchery populations

To investigate the genetic relationship between the Lake Biwa native populations and the captive breeding line that was released into the Lake Biwa watershed, we analyzed 120 native individuals from 14 reference sites, including the Pacific, Sea of Japan, and Lake Biwa watersheds. In addition, 29 stocked individuals were used as representatives of the established stocked population (Table 1). When all individuals were assumed to be within one population (*p* = 1), a total of 103 single nucleotide polymorphisms (SNP) were obtained from the genomic DNA of these tested individuals by MIGseq. The average missing rate was 17.5% per an individual in this initial analysis.

**FIGURE 3:**
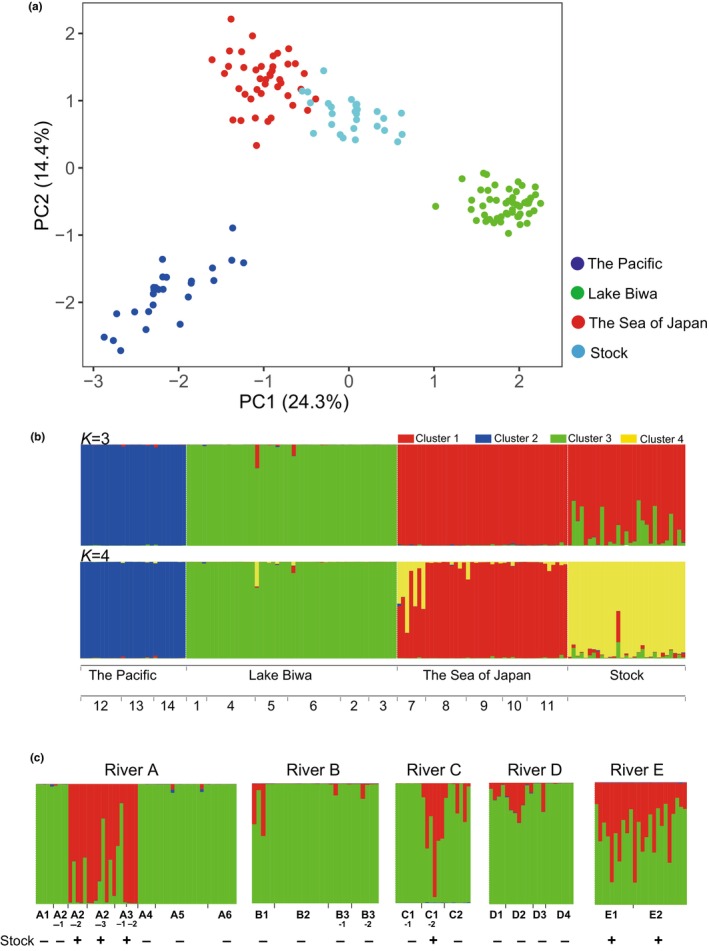
Population genetic structure of white‐spotted charr genetic groups and stocked line in the Lake Biwa watershed. (a) Principal component analysis of individual genotypes. Individuals from each watershed group (the Sea of Japan, the Pacific, and Lake Biwa) are colored red, blue, and green, respectively. Stocked individuals are shown in light blue. Watershed classification is according to the contemporary watershed. (b) Bayesian individual clustering results from STRUCTURE analysis. The STRUCTURE bar plot across individuals from all sampling locations is shown for *K* = 3 and 4. Each vertical line indicates an individual from the sampling sites and a stocked line. Colors represent their inferred ancestries from *K* ancestral populations. Typically, red, blue, and green represent the ancestries of the Sea of Japan, Pacific Ocean, and Lake Biwa genetic clusters, respectively. (c) Individuals from the sampling sites were analyzed using STRUCTURE with the cluster number set to three (*K* = 3) for each test. Bar plots of the tested rivers (A–E) are shown in order.

Principal component analysis (PCA, Figure 3a) clearly showed three genetic groups corresponding to their original (Pacific, Sea of Japan, and Lake Biwa) watersheds. STRUCTURE analysis also represented three distinct genetic clusters corresponding to their original watersheds, when *K* = 3 was assumed (Figure 3b; *K* = 3). On the other hand, the hatchery‐reared individuals were positioned between Lake Biwa and the Sea of Japan watershed groups on the PCA plot (Figure 3a). Consistent with these results, the STRUCTURE analysis clearly showed that the hatchery individuals exhibited admixture, primarily characterized by the genetic background of the Sea of Japan group with traces of the Lake Biwa genetic background, though the ratio of the two varied among individuals (Figure 3b; *K* = 3). Note that all 29 stocked individuals carried the Sea of Japan background in *K* = 3. When *K* = 4 was assumed, the stocked population was shown as a distinct genetic cluster, though the three native genetic clusters (the Sea of Japan, the Pacific, and Lake Biwa) became partially mixed and complicated (Figure 3b; *K* = 4).

Their population genetic statistics were calculated using the 520 SNPs obtained from the actual number of populations used (reference and testing sites, *p* = 34) (Table 1). Native populations in rivers flowing into Lake Biwa generally showed low values in both heterozygosity (*H*_E_, 0.034–0.076) and nucleotide diversity (*P*_i_, 0.03371–0.07735) when compared with the Sea of Japan populations. The Pacific populations also showed low heterozygosity levels comparable to those observed in the Lake Biwa populations (Table 1). The inbreeding coefficient (*G*_IS_) for Lake Biwa, as well as that for the Pacific populations, had a large negative value. These results suggest that native populations in the Lake Biwa and the Pacific watersheds, at least those used in this study, experienced a loss of genetic diversity. The genomic DNA of captive breeding individuals exhibits higher heterozygosity and a lower inbreeding coefficient compared to the values observed in the Lake Biwa native populations (Table 1).

### 3.2 Genetic introgression from the hatchery supplemented line

**FIGURE 4:**
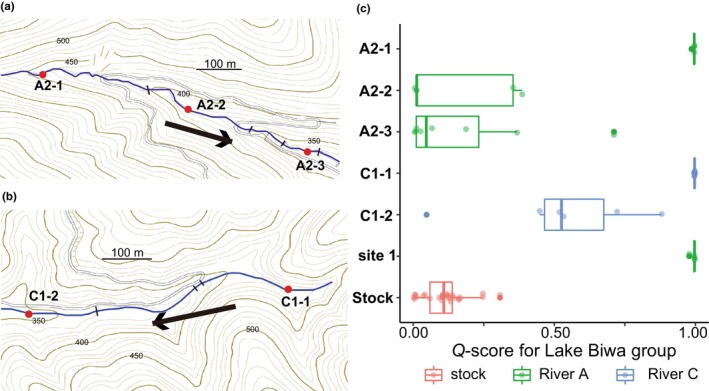
The fine‐scale population genetic structure was examined for sites within the same branches, specifically branches A2 and C1. (a) Detailed map of the experimental sections of branch A2 of River A. A2‐1, A2‐2, and A2‐3 are the upper, middle, and lower parts of the stream, respectively. (b) A map of experimental sections C1‐1 (upper) and C1‐2 (lower) is shown at the same scale as (a). These maps are based on the Digital Topographic Map published by the Geospatial Information Authority of Japan. (c) Box plot of *Q*‐values for the Lake Biwa cluster (cluster 3 in Figure 3b) calculated from the STRUCTURE analysis. A2‐1, A2‐2, and A2‐3 represent sectioned areas of the same branch of River A (green plots), while C‐1 and C‐2 belong to River C (light blue plots). The *Q*‐values of individuals from the typical Lake Biwa native population (Reference site 1) and stocked individuals are also shown (salmon plots). Each dot represents the *Q*‐value of an individual. The lower (Q1) and upper (Q3) quartiles represent the 25th and 75th percentiles, respectively. The diagram also includes the median, depicted as a bold line, and the observed data points representing the *Q*‐scores of the samples. Data points falling outside the range defined by the whiskers (typically 1.5 times the interquartile range from Q1 and Q3) are plotted as outliers (shown by distinct markers).

Testing samples were collected from the five rivers, A, B, C, D, and E, each of which discharges into Lake Biwa (Figure 2). We chose eight sampling sites from the mainstream and six branches of River A (Table 1b and Figure 2b). In branch A2, three sampling sites—upper, middle, and lower parts—which were separated by erosion control dams, were set (sites A2‐1, A2‐2, and A2‐3; Figure 4a). When samples from River A were analyzed with reference native and hatchery fish, individual‐based introgression was visualized as the genetic admixture of the Sea of Japan background in structure analysis, *K* = 3 (Figure 3c and Figure S1a). Individuals in sites 1 (A1), A2‐1, A4, A5, and A6, for which there were no records of stocking, showed a basically pure genetic structure characteristic of the Lake Biwa group (River A; Figure 3c). On the other hand, fish from branches A2‐2, A2‐3, and A3 (A3‐1 and A3‐2) showed an admixture of the Lake Biwa and Sea of Japan groups, which is characteristic of the genetic structure of hatchery individuals (River A; Figure 3c). We quantified the genetic admixture of other clusters in Lake Biwa populations based on their ancestry coefficients (*Q*‐scores) for the Lake Biwa genetic cluster. Populations from branch A2 (Figure 4a) showed different genetic patterns among the three sites, that is, populations from the middle (A2‐2) and lower (A2‐3) parts had a genetic admixture, whereas the population from the uppermost part (above the dam, A2‐1) had the pure Lake Biwa‐type genetic structure (Figure 4a,c).

Since River B was not managed by a fishery cooperative, sites in this river did not receive any official recorded supplementation. Accordingly, the population at site 4 (B2) was free from introgression (River B; Figure 3c). However, the Sea of Japan genetic background was detected in the genomic DNA of individuals from the other three sites, though the admixture ratios varied among individuals (River B; Figure 3c).

Samples from River C were collected at three sites (C1‐1, C1‐2, and C2), two of which were in the same branch (C1‐1 [upper] and C1‐2 [lower]) but were separated by an erosion control dam (Figures 2b and 4b). The hatchery fish had been released around C1‐2 and the region downstream of it. The population at C1‐1 (above the dam) had the pure genetic background of the Lake Biwa type (Figures 3c and 4c), suggesting that the artificial dam prevented an upward gene flow from C1‐2 to C1‐1. A genetic admixture of the Sea of Japan type was seen at site C‐2 (River C; Figure 3c), though no stocking history was seen there.

We collected samples from the four sites of River D (D1, D2, D3, and D4) (Table 2). None of the four sites had an official supplementation history. Slight genetic introgression from the hatchery line (Sea of Japan) was observed in the populations from sites D1 and D2 (River D; Figure 3c). In contrast, all samples from River E showed an admixture similar to the stocked line (River E; Figure 3c and Figure S1e). Both sites of River E had a stocking history.

### 3.3 Relationship between genetic introgression and river conditions

**FIGURE 5:**
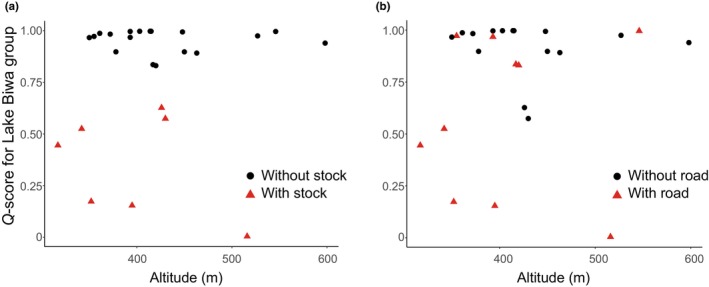
Relationship between genetic admixture and the landscape condition. The average *Q*‐scores for the Lake Biwa type of each tested population as well as the three reference populations (sites 2, 3, and 6) were plotted against their altitudes, focusing on the stocking history (a) and road availability (b).

To assess the relationship between landscape environments and the preservation of the original genotype, *Q*‐scores for the Lake Biwa group were plotted against the altitude of each sampling site, with a focus on the supplementation history (Figure 5a) and the availability of roadways for vehicles (Figure 5b). Multiple regression analysis using explanatory variables of the presence or absence of supplementation history, roadways, and artificial obstacles, indicated that supplementation history (*p* < .001) and road availability (*p* < .05) made significant contributions (Table 3).

**TABLE 3:** Coefficients and statistics of the multiple regression analysis.

	Estimate	Std. error	*t* Value	Pr(>|*t*|)
(Intercept)	1.148	2.131 × 10^−1^	5.387	<.0001
Altitude	4.405 × 10^−4^	3.792 × 10^−4^	−1.162	.259
Stock	−5.237 × 10^−1^	1.336 × 10^−1^	−3.919	<.001
Road	−1.290 × 10^−1^	5.864 × 10^−2^	−2.200	<.05
Isolation	−2.895 × 10^−2^	1.349 × 10^−1^	0.215	.832

*Note*: Coefficients and statistics of the multiple regression analysis for each variable: altitude (m), supplementation history (stock), road availability (road), and isolation by dams and/or falls (isolation).

### 3.4 Differentiation of the populations within the Lake Biwa genetic group

Pairwise *F*_ST_ values among reference populations are shown in Table 4. The *F*_ST_ values of populations in the Lake Biwa watershed are rather closer to the group of the Sea of Japan than that of the Pacific. With regard to pairwise *F*_ST_ within the Lake Biwa group, the values ranged from 0.119 to 0.560 (Table 4), suggesting some regional differentiation in the Lake Biwa genetic group (see below). In contrast, much lower pairwise *F*_ST_ values (below 0.15) were obtained among populations from the sites where local fishery cooperatives released hatchery fish (Table 5).

**TABLE 4:** Pairwise *F*_ST_ values among 14 reference populations from the three major watersheds (Lake Biwa, the Sea of Japan, and the Pacific) and the hatchery‐reared population (Stock).

	Population no.	Lake Biwa						Sea of Japan					Pacific			Stock
		1 (A1)	2	3	4 (B2)	5 (D4)	6	7	8	9	10	11	12	13	14	
Lake Biwa	1 (A1)	0	–	–	–	–	–	–	–	–	–	–	–	–	–	–
	2	0.119	0	–	–	–	–	–	–	–	–	–	–	–	–	–
	3	0.494	0.261	0	–	–	–	–	–	–	–	–	–	–	–	–
	4 (B2)	0.322	0.264	0.346	0	–	–	–	–	–	–	–	–	–	–	–
	5 (D4)	0.560	0.476	0.505	0.303	0	–	–	–	–	–	–	–	–	–	–
	6	0.402	0.327	0.533	0.332	0.139	0	–	–	–	–	–	–	–	–	–
Sea of Japan	7	0.530	0.559	0.672	0.593	0.504	0.521	0	–	–	–	–	–	–	–	–
	8	0.530	0.541	0.570	0.498	0.525	0.548	0.219	0	–	–	–	–	–	–	–
	9	0.566	0.536	0.545	0.461	0.551	0.583	0.224	0.215	0	–	–	–	–	–	–
	10	0.691	0.717	0.698	0.622	0.726	0.674	0.455	0.331	0.372	0	–	–	–	–	–
	11	0.483	0.526	0.556	0.459	0.527	0.540	0.282	0.169	0.211	0.317	0	–	–	–	–
Pacific	12	0.774	0.732	0.817	0.706	0.727	0.647	0.594	0.514	0.588	0.603	0.558	0	–	–	–
	13	0.739	0.784	0.825	0.680	0.711	0.740	0.576	0.493	0.645	0.619	0.518	0.230	0	–	–
	14	0.856	0.808	0.839	0.762	0.750	0.717	0.554	0.540	0.642	0.679	0.598	0.319	0.483	0	–
	Stock	0.179	0.139	0.320	0.229	0.212	0.156	0.227	0.224	0.233	0.244	0.210	0.405	0.360	0.431	0

*Note*: Populations 1, 4, and 5 were also used as tested samples (population numbers as tested samples are shown in parentheses).

**TABLE 5:** *F*_ST_ distances among populations from the branches with recorded stocking in the Lake Biwa watershed.

Pop. no.	A2‐3	A3‐2	C1‐2	E1	E2
A2‐3	0	–	–	–	–
A3‐2	−0.042	0	–	–	–
C1‐2	0.035	0.073	0	–	–
E1	0.063	0.086	0.070	0	–
E2	0.137	0.150	0.131	0.083	0

**FIGURE 6:**
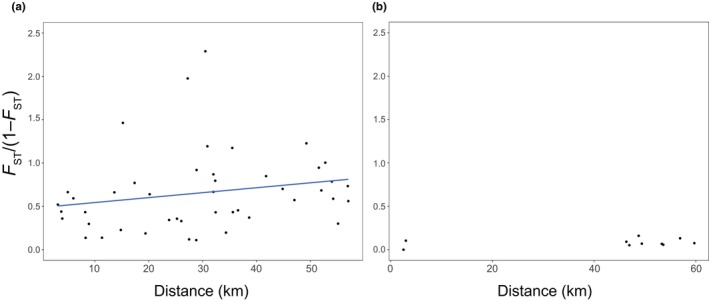
Isolation‐by‐distance (IBD) plots for populations of white‐spotted charr in the Lake Biwa watershed. Pairwise *F*_ST_ values, expressed as *F*_ST_/(1 − *F*_ST_), among the native populations (a) and stocked populations (b) in the Lake Biwa watershed were plotted against direct geological distance. Native populations were selected according to the results of the STRUCTURE analyses, while individuals from the recorded stocked area were used as stocked. A positive correlation was found in the case of native populations. The significance of the correlations was tested by the Mantel test (*p* = .0157, Mantel's *r* = .317 in [a]; and *p* = .200, Mantel's *r* = .250 in [b]).

Correlations between the genetic and geological distances were analyzed for both types of populations, with or without stocking. “With stocking” populations were selected from the sites with recorded official stocking histories, as listed in Table 2. The isolation by distance (IBD) plot for the native populations of the Lake Biwa genetic group showed a significant correlation by the Mantel test (Mantel's *r* = .317, *p* < .05) (Figure 6), whereas that for the population from supplemented sites did not show such a correlation (Mantel's *r* = .200, *p* = .250) (Figure 6).

### 3.5 Population genetic structure of native white‐spotted charr in the Lake Biwa system

**FIGURE 7:**
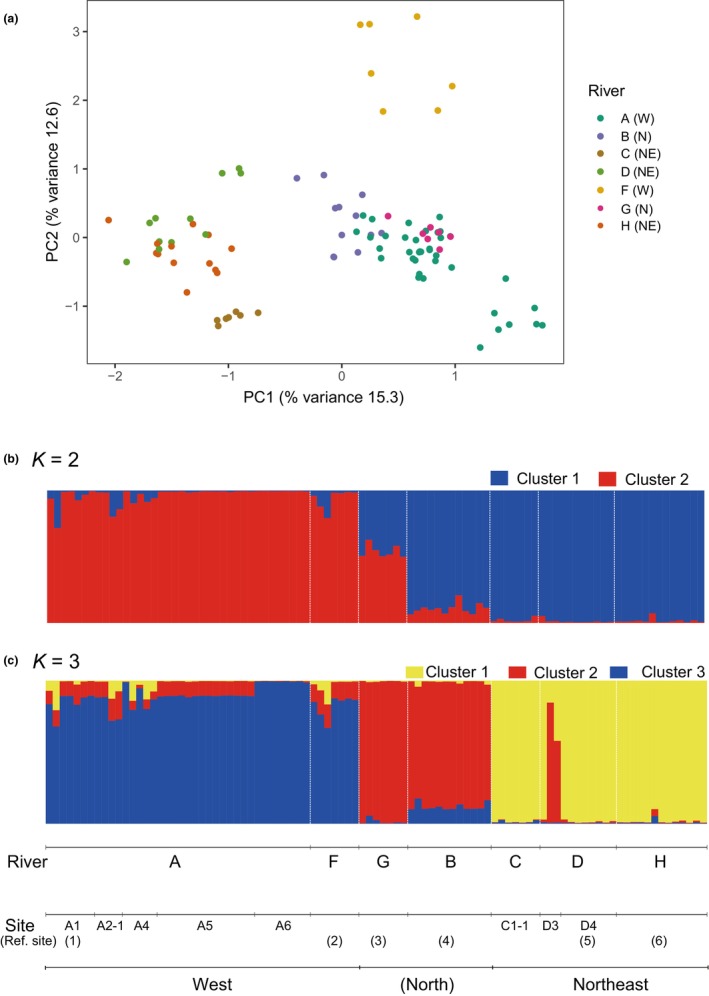
Genetic differentiation of white‐spotted charr in the Lake Biwa catchment. (a) Principal component analysis (PCA) of individual genotypes. Individuals from each river system are indicated by different colors. The location of each site is given in the brackets. W, N, and NE mean west, north, and northeast of the lake. (b) and (c) Bayesian individual clustering results from STRUCTURE analysis. STRUCTURE bar plots were generated using data from individuals across all sampling locations for *K* = 2 (b) and *K* = 3 (c). Each vertical bar in the plot represents an individual from the three watersheds and the stocked population. Different colors are used to indicate the assumed two or three clusters.

To obtain the population genetic structure of the Lake Biwa group, PCA and STRUCTURE analysis were carried out on white‐spotted charr individuals in populations that were assumed to be native (populations from reference sites 1 [A1], 2, 3, 4 [B2], 5 [D4], and 6), and from tested sites (A2‐1, A4, A5, A6, C1‐1, and D3) (Figure 3). The PCA plot shows that PC1 well resolves the western (Rivers A and F) and northeastern (Rivers C, D, and H) clusters (Figure 7a). The number of clusters was assumed to be two or three (Figure S2) according to the Evanno method (Evanno et al., 2005). When *K* = 2 was adopted, the Lake Biwa native populations could be divided into two clusters, the western and northeastern, by STRUCTURE (Figure 7b). When *K* = 3 was assumed, the tested populations were classified into three genetic clusters, each of which was composed of the western rivers (Rivers A and F), the northern rivers (Rivers G and B), and the northeastern rivers (Rivers C, D and H) (Figure 7c). The western part (Rivers A and F) and the northeastern part (Rivers C, D, and H) were apparently differentiated, while the northern part (Rivers G and B) was intermediate between the other two (Figure 7). In conjunction with the results of the PCA, it was observed that the western, northern, and northeastern populations within the Lake Biwa group exhibited genetic differentiation, and a genetic cline was observed in the northern population, showing some similarity to the western and northeastern group (Figure 7).

## 4 DISCUSSION

This study revealed the genetic impact of the stocked strain on the differentiated native populations in a fine‐scale manner using a simplified design of white‐spotted charr in the Lake Biwa basin. Supplementation with hatchery‐reared fish has been one of the simplest methods to complement decreasing populations of freshwater fish such as charr. However, the direct effects of supplementation on the restoration of endangered populations are still controversial (Araki et al., 2008; Narum et al., 2017; Terui et al., 2023; Willoughby & Christie, 2019). Genetic introgression of stocked fish would lead to a loss of locally differentiated genetic variation, ultimately resulting in the depletion of the native local population itself (Lamaze et al., 2012; Marie et al., 2010; Willoughby & Christie, 2019). A previous study suggested that the Lake Biwa group constitutes a distinct genetic group that is discernable from the Pacific and Sea of Japan groups (Masuda et al., 2023). On the other hand, we showed here that the hatchery line was an admixture composed mainly of the Sea of Japan genomic background along with traces of the Lake Biwa genomic backgrounds, though the ratios of the two backgrounds varied among individuals. This design enabled us to detect the hatchery introgression as an admixture of the Sea of Japan genetic background into the native populations throughout the entire Lake Biwa drainage area.

The supplementation history and road availability well explained the occurrence of genetic admixtures, regardless of the altitude. In accordance with this result, most stocking attempts are done by using vehicles. However, some exceptional cases of stocking were seen in River E, where releasing has been carried out by volunteers on foot. These attempts would allow gene flow to areas where no roadway is available. Individuals from the River E sites generally had higher ratios of the native Lake Biwa genetic background than the hatchery individuals, despite annual stocking (Figure 3c; River E). This implies that these sites had been the habitats of native individuals. Slight admixture patterns of less than approximately 15% were observed at the non‐stocking sites in Rivers C and D (Figure 3c; Rivers C and D). These admixture patterns may have been caused by small unrecorded quantities of releases and/or the relatively long time that had passed since the last supplementation was conducted at these branches. Létourneau et al. (2018) predicted the genetic impact of stocking on the wild populations of brook charr (*S. fontinalis*) by combining genotyping by sequence and modeling. They found that domestic alleles which are unfit for the local environment could be purged through time by a selective process and genetic drift after stocking cessation when the introgressive hybridization rates are low (ex.: <0.35) (Létourneau et al., 2018). Another case study using marble trout, a local variant of brown trout (*Salmo trutta*), observed that introgressive hybridization with supplemented hatchery trout is gradually reduced and almost cleared on the genome (<2%) within 20 years, suggesting that the native genetic structure is restored after cessation of supplementation (Berrebi et al., 2022). Therefore, it is possible that these genetic patterns observed in Rivers C and D of the current study could revert to a more native‐like state if stocking supplementation were to be discontinued. Although no fishery cooperative manages River B, genetic introgression of the Sea of Japan type was detected in several sites. Two possible causes can be assumed: (1) unrecorded release of hatchery fish, and (2) geological causes such as stream capture. Concerning the first possibility, site B3‐2 was easily accessible because it was along a main paved road. In contrast, site B3‐1, which was upstream of B3‐2, was away from the road and separated from site B3‐2 by a dam. The possibility of unrecorded release at site B3‐1 cannot be ruled out, but the second possibility is also plausible, particularly since it has been posited that this site was once part of the river flowing into the Sea of Japan before a stream capture event (Matsu'ura, 1999). If the part of River B that included the B3 sites had been a part of the north‐flowing river discharging into the Sea of Japan, white‐spotted charr in these sites (B3‐1 and B3‐2) would have the Sea of Japan genetic background. Indeed, individuals from B3‐1 and B3‐2 showed an admixture containing a trace of the Sea of Japan's genetic background (Figure 3c) that could have been caused by gene flow from the individuals in the captured area. Further examination in other branches adjacent to the B3 sites and using another species is required.

As described above, white‐spotted charr populations in the Lake Biwa drainage area constitute one local genetic group, which is the southernmost genetic group of white‐spotted charr within its continuous distribution range. Authentic southernmost populations remain discontinuously in a few isolated habitats in the Kii Peninsula. They are currently under a human‐mediated management scheme to conserve the remaining local populations (the Pacific group, Kirikuchi [Sato, 2006; Sato & Harada, 2008; Yamamoto et al., 2019, 2023]). The Lake Biwa type can be regarded as an evolutionarily significant unit (Fraser & Bernatchez, 2001). Despite the significance of the local group, habitats of native white‐spotted charr in the Lake Biwa watershed were restricted to the upstream region, where no supplementation had occurred, and their genetic diversity appeared to be declining when compared with the populations with stocking. Regarding the native populations, they exhibited genetic differentiation within the Lake Biwa group (Figure 7). For example, one group is located in the western region and comprises Rivers A and F, while the other group is in the northeastern region and consists of Rivers G, B, C, D, and H when two ancestral groups are assumed (*K* = 2) (Figure 7b). When *K* = 3 was adopted in the STRUCTURE analysis, the northern group (Rivers G and B), which showed a genetic structure intermediate between those of the western and northeastern groups in *K* = 2 (Figure 7b), appeared to be differentiated from these other two groups (Figure 7c). A previous study suggested that populations inhabiting the eastern part of the drainage area are differentiated from other rivers in the drainage area (Kikko, Kai, et al., 2008; Kikko, Kuwahara, et al., 2008). Further genetic differentiation would be possible within the Lake Biwa group, and this is currently under investigation. Native populations in the watershed showed significant IBD (Figure 6a), whereas IBD was not significant among stocked branches. This suggests that genetic differentiation was largely reduced by hatchery‐line supplementation (Figure 6b and Table 5). The significance of IBD among the native Lake Biwa populations implies past dispersal and gene flow among these populations, likely via Lake Biwa, whereas the insignificance of IBD among populations with hatchery supplementation clearly indicated that conventional stocking did not contribute to the maintenance of genetic diversity in locally differentiated populations, even though it increased heterozygosity in those populations.

The southernmost and low‐altitudinal distribution is indicative of high‐temperature adaptation in the genetic group. During the current Anthropocene period, global warming induced by climate change is seriously affecting the planet's ecosystems (Hoffmann & Sgró, 2011). Global warming poses a critical challenge to entire freshwater ecosystems (Brauer & Beheregaray, 2020; Ricciardi & Rasmussen, 1999), and among the animals within these ecosystems, coldwater‐adapted species are considered to be the most vulnerable to rising temperatures (Booher & Walters, 2021; Eby et al., 2014; Kovach et al., 2017; Laulhère et al., 1992; McCullough et al., 2009; Nakano et al., 1996; Wenger et al., 2011; Wu et al., 2023). Since a small branch acts as a source or sink of local populations of such cold‐water specialists within the temperate Japan archipelago (Tsuboi et al., 2022), the small local populations, such as those described in this study, should be conserved. To preserve the adaptive genetic variation and restore genetic diversity in isolated native populations, it may be necessary to facilitate artificial gene flow through methods such as translocation, reintroduction, and captive breeding, using regional native individuals (Flanagan et al., 2018; Meek et al., 2023; Sato & Harada, 2008). A recent study on the reintroduction of brook trout suggested the importance of incorporating genomic and demographic information in selecting populations for reintroduction; this approach helps in the preservation of genetic diversity and locally adaptive variations (White et al., 2023). In this context, conducting further genomic studies is crucial for providing the information necessary to determine the possible conservation unit of the white‐spotted charr genetic group in the distribution margin.

Recent studies have suggested that local populations with adaptive genetic variation will be crucial in our response to environmental change (Meek et al., 2023; Razgour et al., 2019; Savolainen et al., 2013; Tsuboi et al., 2022). However, smaller populations in more isolated habitats are theoretically subject to a greater risk of extinction (Boyce, 1992), as also suggested by our present study. Our findings will provide insight into the status and consequences of human‐mediated conservation performance on a regional genetic group of a coldwater specialist species in its warm range margin. However, this case study has broader implications beyond the study of a single regional species. Effective supplementation and reintroduction that contribute to the conservation of locally adapted genetic variations will be enabled by further empirical, experimental, and theoretical researches.

## AUTHOR CONTRIBUTIONS

**Taro Masuda:** Conceptualization (lead); data curation (lead); formal analysis (equal); investigation (lead); methodology (equal); project administration (lead); resources (lead); visualization (equal); writing – original draft (lead). **Yoshiko Shimono:** Conceptualization (supporting); data curation (supporting); formal analysis (supporting); investigation (supporting); methodology (equal); supervision (equal); validation (supporting); visualization (supporting); writing – original draft (supporting); writing – review and editing (supporting). **Daisuke Kishi:** Investigation (supporting); resources (supporting); writing – original draft (supporting); writing – review and editing (supporting). **Itsuro Koizumi:** Conceptualization (supporting); data curation (supporting); formal analysis (equal); investigation (supporting); supervision (equal); writing – original draft (supporting); writing – review and editing (supporting).

## CONFLICT OF INTEREST STATEMENT

We declare we have no conflict of interests.

## Supporting information


Figure S1.
Figure S2.

## Data Availability

The raw sequence data were deposited in the DDBJ Sequence Read Archive Database (Accession number: DRA017376).
